# Zero→Two-Dimensional Metal Nanostructures: An Overview on Methods of Preparation, Characterization, Properties, and Applications

**DOI:** 10.3390/nano11081895

**Published:** 2021-07-23

**Authors:** Ming Yang, Xiaohua Chen, Zidong Wang, Yuzhi Zhu, Shiwei Pan, Kaixuan Chen, Yanlin Wang, Jiaqi Zheng

**Affiliations:** 1 School of Material Science and Engineering, University of Science and Technology Beijing, Beijing 100083, China; m_young90@163.com (M.Y.); zhuyuzhi@ustb.edu.cn (Y.Z.); chenkxustb@126.com (K.C.); wangyanlin921@ustb.edu.cn (Y.W.); 18710252086@163.com (J.Z.); 2 State Key Laboratory for Advanced Metals and Materials, University of Science and Technology Beijing, Beijing 100083, China; panshiwei77@sina.cn

**Keywords:** metal nanostructures, preparation, characterization, properties, applications

## Abstract

Metal nanostructured materials, with many excellent and unique physical and mechanical properties compared to macroscopic bulk materials, have been widely used in the fields of electronics, bioimaging, sensing, photonics, biomimetic biology, information, and energy storage. It is worthy of noting that most of these applications require the use of nanostructured metals with specific controlled properties, which are significantly dependent on a series of physical parameters of its characteristic size, geometry, composition, and structure. Therefore, research on low-cost preparation of metal nanostructures and controlling of their characteristic sizes and geometric shapes are the keys to their development in different application fields. The preparation methods, physical and chemical properties, and application progress of metallic nanostructures are reviewed, and the methods for characterizing metal nanostructures are summarized. Finally, the future development of metallic nanostructure materials is explored.

## 1. Introduction

**Figure 1:**
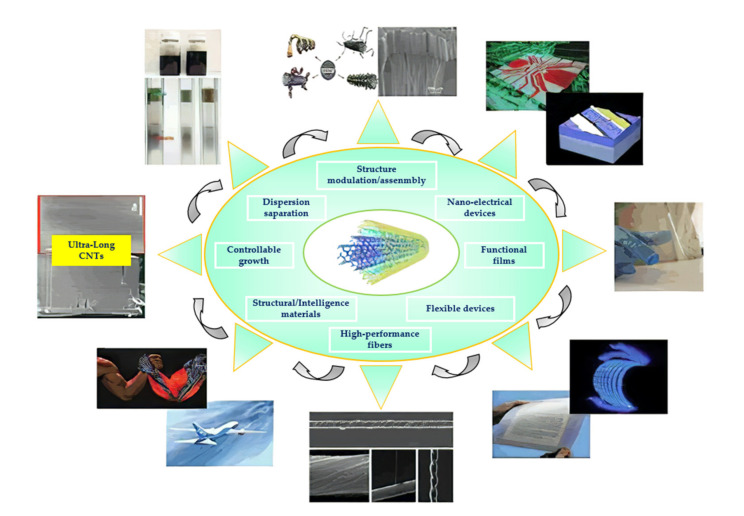
Various applications of nanomaterials.

Nanomaterial has been a much-researched field during the past decade. Normally nanomaterials were prepared by various methods in the laboratory. Nanomaterials were investigated, from the difference between nanomaterials and ordinary materials, to the special properties of nanomaterials themselves; from the preparation of single nanomaterials to the preparation of composite nanomaterials. Due to the superior properties of nanomaterials in terms of force, heat, light, electricity, and magnetism, nanomaterials have a wide range of applications, including medical, home appliances, machinery, and electronic products (Figure 1).

According to different application fields, nanomaterials can also be divided into nano-optical materials, nano-magnetic materials, and nano-semiconductors. Although nanomaterials have been widely used to a certain extent, how to controllably fabricate nanomaterials with characteristic microstructures and peculiar physical properties is still particularly important [1], which is the key to determining application fields of the nanomaterials.

Nanomaterials have been widely investigated in modern physics, chemistry, materials science, and life sciences, and the research results have gradually been widely used in high-tech fields, such as modern microelectronics, mesoscopic nanoelectronics, and molecular electronics [2], etc.

Common nanostructures include nanowires, nanorods, nanoribbons, nanoblocks, nanoparticles, and nanotubes. Among them, nanoparticles have many forms [3], such as polyhedrons, plates, prisms, rods, wires, nanoboxes, nanocages, dumbbells, nano spacecraft, stars, branches and wires, tree branches, nano rings, etc. This kind of nanostructure has a series of unique physical and chemical properties and a wide range of application prospects, including traditional catalysis, electronics, photography, information storage, etc., as well as new applications, in photonics, sensing, imaging, and medicine, which creates strong interest in its structural characteristics, growth mechanism, and potential applications. Nanostructured materials are categorized into metal nanostructured materials and non-metallic nanostructured materials (such as nanoclays natural [4,5], etc.). This article mainly explores metal nanostructured materials (such as nano-gold, nano-silver, nano-copper, etc.).

Metal nanostructures have drawn widespread attention due to their interesting characteristics and potential technical application value [6,7,8,9,10,11,12,13,14,15]. Metal nanocrystals are determined by a series of physical parameters, which include their size, shape, composition, and structure. In principle, people can control any of these parameters, but the flexibility and range of changes are highly sensitive to specific parameters. For example, in the case of local surface plasmon resonance (LSPR), surface-enhanced Raman scattering (SERS), calculations, and experimental results show that the shapes and structures of gold and silver nanocrystals play the most important role in determining the number, position, and intensity of LSPR modes, as well as the spectral region or the use of SERS [16] for effective molecular detection. In the case of catalysis, it is the activity of metal nanocrystals that can be enhanced by reducing the size [17]. However, the most sensitive to selectivity is the accumulation of atoms on the surface or exposed surface nanocrystals [18]. For example, Pt can selectively catalyze different types of chemical reactions on {100} and {210} crystal faces the most active reaction to H_2_ and CO [19]. There are many other examples that clearly illustrate the importance of shape control for the effective use of metal nanocrystals. Researchers have prepared and characterized different metal nanostructures through a variety of physical and chemical methods, to explore their valuable properties. Among them, the physical preparation method is vacuum deposition [20,21], electron beam lithography [22,23], and laser etching [24,25,26], etc. Nanostructures prepared by such methods are usually attached to various substrates while metal nanostructures synthesized by various wet chemical synthesis methods, such as nanospheres, nanorods, nanocubes, nano double cones, and core-shell nanocomposite structures usually rely on the active agent molecules adsorbed on their surfaces to maintain a stable and monodispersed state in the solution [9,10,11,12]. In recent years, researchers have conducted in-depth studies on the optical properties of metal nanostructures through a series of optical characterization techniques such as dark field [27,28,29,30], near field [31,32,33], confocal microscopy [34,35,36], as well as spectroscopy and numerical electromagnetic simulation tools [37,38,39]. They found a new phenomenon related to LSPR, which reveals that single and assembled metal nanostructures contain the basis of abundant photon-photon, photon-electron, and electron-electron interactions physical phenomenon. As a result, metal nanostructures are widely used in various fields with their unique electromagnetic properties, such as bio-cell label [40], sensing [41,42,43,44], as well as data storage and optoelectronics [45,46,47]. In addition, metal nanostructures assembled into various special structures have special research and application values due to the coupling effect of surface plasmon resonance [48,49,50]. A classic example is the metal nanostructures coupled with SERS, which effectively amplifies the Raman scattering signal (the amplification effect can currently reach 10^8^–10^10^ orders of magnitude) due to the greatly enhanced local electric field. Therefore, we can more clearly detect the Raman signal of a single molecule [50].

## 2. Fabrication Methods

The performance of metal nanostructures significantly depends on size, shape, and aspect ratio, which has prompted people to research and develop preparation techniques that can better control the shape and size of nanostructures. At present, the preparation technology of metal nanostructures mainly includes a chemical method, template method, photolithography technology, self-assembly of metal nanoparticles, and microbial-assisted synthesis technology.

### 2.1. Chemical Synthesis

Chemical synthesis is currently one of the most important methods for preparing nanometals, but the preparation process is not easy to control and is limited by the types of precursor compounds that can be selected. Chemical synthesis methods can be further divided into: The chemical reduction method, seed growth method, and diffusion method.

The most commonly used and mature method for the synthesis of bimetallic nanoparticles is chemical reduction. In this method, the main principle is that the metal salt (such as chloroauric acid) in the solution is reduced by the reducing agent. When synthesizing bimetallic nanoparticles, we need to use a reducing agent to reduce two different metal ions, so this method is also called a co-reduction method. Metal ions with a higher reduction potential are firstly reduced to nuclei, and other metal ions are subsequently reduced and precipitated on the surface of the core to form a shell layer, thereby obtaining a bimetallic core-shell structure. In order to better control the reduction and nucleation process, appropriate surfactants or polymer ligands are used to passivate the particle surface during the preparation process, and the reduction step is controlled or a relatively mild reducing agent is used. NaBH_4_ is a commonly used strong reducing agent. In many works, it is used to synthesize bimetallic nanostructures for example, the successful preparation of Ag@Cu [51] and Au@Pd [52] bimetallic nanostructures. The weak reducing agent can be AA, H_2_, or CO [53,54], etc.

**Figure 2:**
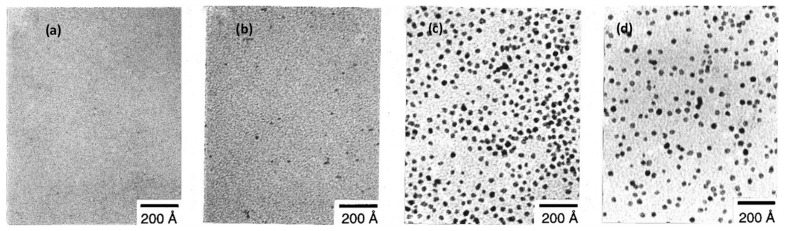
Pt nanoparticles of different diameters prepared by chemical methods: (**a**) 10 min, (**b**) 20 min, (**c**) 30 min, and (**d**) 180 min. (PVP/Pt = 10, [methanol] = 90 vol %). Reprinted with permission from Ref. [55]. Copyright 1999 American Chemical Society.

Generally speaking, the metal salt precursor is mixed with a reducing agent in the presence of a stabilizer to achieve the purpose of controlling the size and shape of the metal nanostructure. Taking Ag as an example, studies have shown that silver nitrate, as a precursor, has been widely used due to its low price and easy availability. Various reducing agents such as sodium citrate, sodium borohydride, and alcohols are widely used to reduce the metal/silver ions in the solution to metal/silver atoms, which combine to form aggregates and finally form nanostructures. For example, T. Teranishi [55] uses a silver nitrate precursor, combined with an electrophoretic deposition to control the size of monodisperse platinum nanoparticles, as shown in Figure 2.

**Figure 3:**
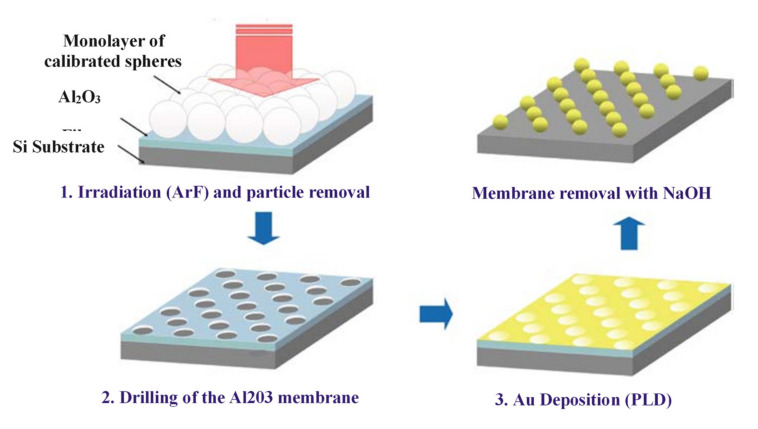
Preparation of ordered Au nanodot arrays by laser pulse irradiation. Adapted from [62], under the terms of the Creative Commons CC BY license.

On the other hand, the photochemical synthesis method combines chemical methods and illumination methods, utilizing a variety of illumination methods to synthesize metal nanostructures. For this technology, light is used to control nanostructures. For example, laser ablation or direct laser irradiation of a metal salt aqueous solution in the presence of a surfactant to prepare a specific shape and size of metal nanoparticles [56,57]. Lasers are also used to modify metal nanoparticles, such as silver nanospheres and silver nanoplates [58,59,60,61], by simple melting, which is called a light-cutting process. Laser-mediated synthesis technology has also achieved success in the preparation of high-quality, controllable metal nanostructures. For example, Kabashin [62] used pulsed lasers for nanomanufacturing. First, a single layer of nanospheres was deposited on an aluminum oxide film, and then irradiated with a single laser pulse. The near-field enhancement under the spheres resulted in parallel nano-drilling of the film. The metal was then deposited and the aluminum oxide film was dissolved in an alkaline solution. Finally, an ordered array of Au nanodots was obtained on the silicon substrate, as shown in Figure 3.

The seed growth method is another commonly used chemical synthesis method with simple operations. The microstructure of metal nanomaterials can be accurately adjusted by changing thermodynamic/kinetic parameters. When the surface of the seed metal is deposited by another metal, a core-shell structure will be formed. When other metals are deposited on specific parts of the seed, a heterogeneous structure will be formed. With previous research, the Au@Ag core-shell structure [63], dendritic Au/Ag bimetallic NPs [64], and bimetallic Cu-Pt alloy nanoparticles with a polyhedral, star, or dendritic shape [65] have been synthesized by the seed reduction method. The diffusion method is a kind of chemical synthesis method for the gentle preparation of bimetallic nanostructures. It has the advantages of good spatial uniformity, mild experimental conditions, and easy accessibility. Zhang et al. [66] used the diffusion method to synthesize octahedral Au/Pd metal nanoparticles. During the diffusion preparation process, the temperature has a significant influence on the synthesis mechanism [67].

The chemical synthesis method can generally prepare nanostructures in batches and large scales. It is simple and easy to implement, however there are harsh requirements on the type of materials and there is difficulty in controlling the structure. It is generally easy to synthesize nanoparticles, but there are certain challenges in the synthesis of other structures.

### 2.2. Template Method

**Figure 4:**
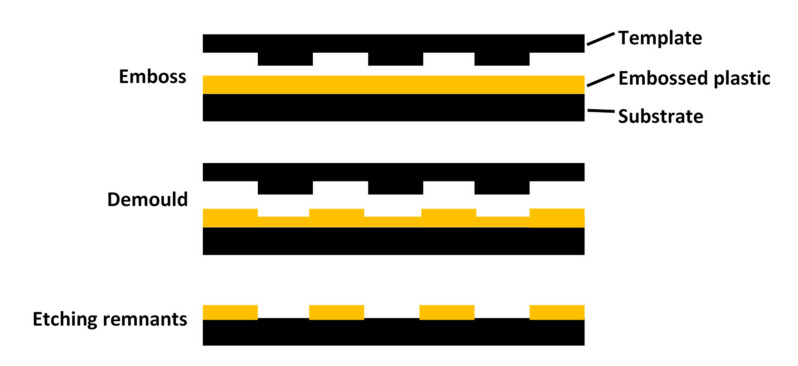
Flow chart of nanoimprint technology.

Nanoimprint lithography, first developed by a fellow of the American Academy of Engineering, Princeton University, Chou, et al. [68] has been proven to be one of the most promising next-generation lithography technologies for nano-scale large-area structure replication. A typical process is shown in Figure 4: First heat the polymer to above its glass transition temperature, then press the polymer into a hard mold with surface microstructure, and finally remove the mold to complete the microstructure of the mold surface to the polymer surface copy transfer. The specific operation steps are shown as follows: (1) Preparing the nano template material; (2) modifying the surface of the template material; (3) coating the selected materials or their precursors on the nano template; and (4) removing the nano template.

The remarkable feature of nanoimprinting is that the resolution is no longer limited by the optical diffraction limit but determined by the pattern size of the mold. Furthermore, nanoimprinting has the advantages of high replication accuracy, high preparation efficiency, low cost, and a simple process. However, this technology is currently mainly applied to polymers and amorphous metals with a lower glass transition temperature [69], and its mechanism is to utilize the characteristics of the flow resistance (indicated by the viscosity coefficient) of the above-mentioned materials that rapidly decrease as the temperature increases. Generally, when an amorphous material is heated to above its glass transition temperature, its viscosity-temperature relationship conforms to the following law [70]:

(1) $$\eta =A\cdot \exp\left(\frac{B}{T-T_{0}}\right)$$

where *η* is the viscosity coefficient, and *A* and *B* are constants independent of temperature. Although increasing the temperature can reduce the flow resistance of the material and facilitate forming, on the other hand, because the amorphous material is in the thermodynamic metastable state, the amorphous material will relax to its more thermodynamically stable crystalline state after experiencing finite time at a high temperature. The stable crystalline state fundamentally limits the nanoimprinting time of amorphous materials, thereby limiting the aspect ratio of the produced nanostructures.

The template method to prepare nanostructures has good controllability. This method has been used to prepare nanostructures that are highly dependent on the specific shape and size of the selected template [71].

There are currently two types of template synthesis methods. One is soft templates, and the other is hard templates. Surfactant molecules are usually formed as soft templates under the critical micelle concentration. This kind of soft template is easy to prepare and does not require complicated technology, but it also has great shortcomings. The stability of the template is poor, the scope of application is limited, and the requirements for materials are relatively strict. Generally speaking, the structure of micelles and reverse micelles in the solution determines the shape and size of the final product.

The most commonly used hard template is the porous anodic aluminum oxide (AAO) template. The high-order nature of the porous structure of the anodic aluminum oxide membrane (AAM) (composed of closely arranged cylindrical hexagonal cells, each cell contains a central cylindrical hole perpendicular to the surface), making it an ideal template for preparing nanostructured materials, suitable for optoelectronics, sensors, magnetic memory, and electronic circuits [72]. The main advantage of the hard template is that it requires less material. Generally speaking, it can mold materials that are softer than its own and has good regularity. Since AAM morphological parameters (film thickness, pore size, and density) can be easily controlled by adjusting the anodizing conditions (voltage and time, anodizing bath composition, and temperature) [73], many studies have shown that template-assisted methods have been used to prepare metal nanostructures of various shapes and sizes, such as silver nanowires [74,75,76], nanopillars [77,78,79], as well as hollow spheres and nanoplates [80,81,82,83,84], etc.

A more common application is the template method combined with an electrochemical deposition to prepare metal nanowires, which are multilayer nanowires grown by switching electrodeposition potentials between two values or by precisely controlling the time of each potential to ensure a constant layer thickness, using constant current growth to prepare heterogeneous alloy nanowires [85]. Nanowires prepared by electrochemical deposition can perfectly replicate the template structure but have poor applicability and strong dependence on chemical reagents. Therefore, most of the metals prepared by this chemical deposition method are inert metals, such as gold, silver, etc. For example, Ching [86] used polycarbonate film and anodic aluminum oxide film as growth templates to successfully prepare gold nanowires under electrochemical deposition conditions.

In addition to manufacturing nanostructures for basic research, template deposition has many technical applications, such as the LIGA (lithographic electroplating) process based on deep X-ray lithography [87], which is used to produce metal structures for microelectromechanical applications. The system may be used as a mold for manufacturing plastic parts, as well as a process used to produce coils and other magnetic recording film sensor head components [88]. Templates suitable for the electrodeposition of metal nanostructures can be prepared by patterning the resist layer using photolithography (optical, X-ray, electron beam, and scanning probe). In this case, the exact shape and location of each feature are clearly determined in advance, or by using random or self-organizing processes to create nanoporous membranes. Examples of nanoporous films that have been successfully used for template electrodeposition include polycarbonate films prepared by etching damage trajectories caused by high-energy particles, and aluminum oxide films prepared by controlled anodization of aluminum. Schwarzacher [85] et al. showed three new examples of template electrodeposition: The growth of hetero-alloy nanowires, the growth of epitaxial multilayer dot arrays, and the growth of superconductor nanowires with controlled microstructures. Each of these examples takes advantage of one or more specific advantages of template electrodeposition technology, namely the ability to prepare nanostructures with controlled crystal structures and/or extremely high aspect ratios.

The characteristic of the template method is that the template can provide geometric constraints, so that the material can grow or shape according to the structure of the template. Most of the nanostructures prepared by the template method are nanowires, which are determined by the particularity of the template. The challenge of preparing the nanostructures by the template method lies in the processing of the template. In addition, the template method to prepare metal nanostructures generally needs to be combined with chemical deposition, but chemical deposition requires harsh material types. At present, it is difficult to realize the chemical deposition synthesis of various metals or alloys. Therefore, the template method still has challenges in the nanosynthesis of metals.

### 2.3. Photolithography

The current top-down nanofabrication technology is actually a nanoscale pattern transfer [89]. Since the vigorous development of nanolithography in the 1980s, many nanolithography technologies have been developed, among which the most advanced electron beam lithography technology is the most widely used for patterning microstructures or systems. Electron beam lithography technology has the unique advantages of high feature size resolution, high processing reliability, high positioning/alignment accuracy, and high pattern replication flexibility. Nanolithography is a process of producing or imprinting nano-sized patterns on a specific substance or substrate as a whole. This branch involves the research and application of nano-processing or nano-patterning of 1-nm nanoparticles [90]. Photolithography technology is mainly divided into two types according to the use of masks or templates. They are masked and maskless lithography. In the mask method, a mask with a desired projection or groove pattern is used to transfer the pattern on the substrate material. This realizes a pattern manufacturing capability with high throughput. On the other hand, maskless lithography, such as electron beam lithography, focused ion beam lithography, and scanning probe lithography, produce irregular patterns by continuous writing without using a mask. Nanolithography technology has made revolutionary contributions in the fields of computers and the Internet and plays a pivotal role in the semiconductor and IC industries [91].

At present, the controllable preparation of metal nanostructures still relies on advanced nanolithography technology, such as electron beam lithography [92]. The application of this technology can produce uniform and regular metal nanostructures. However the yield efficiency is low and the cost is high, especially for the preparation of nanostructures with a high aspect ratio.

The manufacturing range of photolithography is generally from 1 to 100 nanometers. Unlike other processes, photolithography provides a controllable “engraving” capability for metal nanostructures [93]. Researchers have proposed a variety of lithography techniques, such as optical lithography (OL), multiphoton lithography (MPL), scanning probe lithography (SPL), and particle beam lithography (PBL). Particle beam lithography (PBL) uses high-energy particles instead of light beams to produce high-resolution nanostructures. Electron beam lithography (EBL) [94] and focused ion beam (FIB) are the two main subcategories of particle beam lithography, which are integrated with electrons and ions, respectively. Particles with higher energy (>2 KeV) and nanometer-scale wavelengths can reduce diffraction, which limits the resolution of photolithography, so resolutions below 10 nm can be achieved in PBL [95,96]. Compared with photolithography, another advantage of particle beam lithography is that it is a direct writing method without a mask and has excellent flexibility in feature design [95].

**Figure 5:**
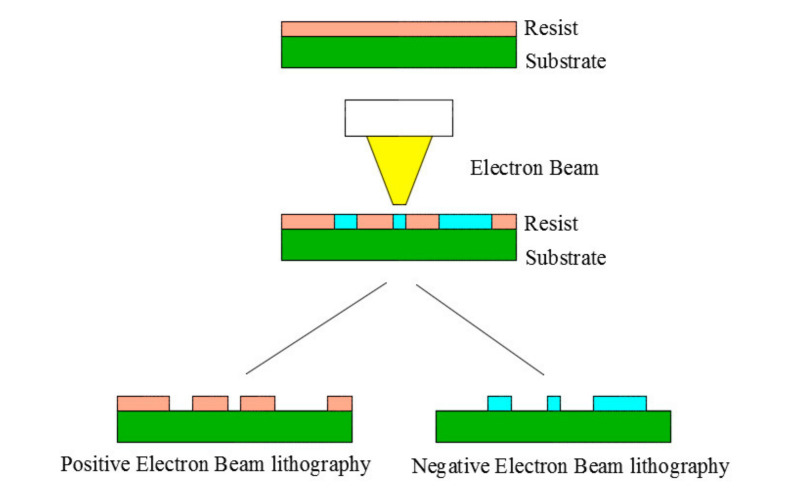
The principle of electron beam lithography. Adapted from [101], under the terms of the Creative Commons CC BY license.

EBL has a lower proximity effect and higher resolution and flux [97]. Electron beam can sweep across the substrate to image the surface. Moreover, it can be used to make resist deposited on the substrate. Due to the low electron energy, EBL can produce polymer resists such as PMMA, PEG, and PAA. As shown in Figure 5, depending on the type of mask, electrons can be cross-linked to produce negative-mode lithography, and degradation can be used to produce positive-mode lithography [98]. The developer is used to remove bad areas. EBL can produce features of various sizes (1 mm–10 nm) on a considerable area. Considering the size and scattering area of the resist molecules, myriads of secondary electrons can affect other areas on the resist. The resolution depends on the molecular size of the resist, scattering range, and secondary electrons. The secondary electrons that are backscattered can affect other areas on the resist. As shown in Figure 5, the positive tone is suitable for the production of nano-patterns with different geometric shapes by coating the peeling area with electron beam evaporation coating technology [96]. For example, EBL technology can be used to deposit silver nanostructures on the substrate to produce nanostructures [99]. Seong [94] used photolithography technology to successfully prepare Ag nanostructures on a glass substrate. They first spin-coated the silver film on the glass substrate, and then used electron beam irradiation to prepare silver nanostructures on the substrate. Tsigara et al. [100] used photolithography to prepare Pt and Ag microelectrodes patterned by hexagonal nanotriangular Ag arrays, and introduced Ag and bimetallic Ag into the Pt microelectrode nanostructure system.

FIB is a high-resolution manufacturing method (10–30 nm) for imaging and manufacturing different materials (such as polymers, silicon wafers, and metals). The principle of FIB is the same as that of scanning electron micrograph (SEM). The difference is that when using SEM and EBL, ions including Ga^+^, Ne^+^, or He^+^ are used instead of electrons [96]. The interaction volumes of gallium, electrons, and helium are different. The interaction volume associated with the secondary electrons (SE2) generated by the focused He+ beam is much smaller than that of e^-^ and Ga^+^, which means that helium ion microscope (HIM) has more advantages for imaging resolution than SEM and Ga^+^-FIB. On the other hand, Ga^+^ has high quality and can produce considerable scattering on the surface. HIM can also scatter the surface at a rate of 30 KeV, and because of the low quality of He+, it is advantageous to make small features at the sub-nanometer level [102]. However, the manufacturing throughput of FIB is less than that of EBL, and there is no need to cover and provide. Due to its low interaction area, FIB becomes a high-resolution manufacturing method. In addition to the semiconductor industry, FIB is also widely used in different scientific applications such as biological materials and biological substances. In biological applications, cells, biological materials, and their interfaces can be visualized, analyzed, milled, and prepared for further technologies (such as TEM) [103]. A FIB instrument consists of a sample table, vacuum chamber, liquid metal ion source, ion column, detector, and gas delivery system [104]. Although FIB milling has a lower proximity and higher sensitivity than electron beam lithography, the large diameter of the Ga^+^ ion beam leads to a low resolution, which causes surface damage and pollution from ion implantation [97].

The biggest advantage of lithography technology is high precision and strong controllability. The wavelength used is generally 0.1–4000 Å, which is generally used as a technical means for precision processing. The disadvantages are also obvious. This technology is inefficient and expensive, making it difficult to be used on a large scale. Recently, nanolithography and its technology have almost surpassed all the obstacles of previous years, and they are changing with each passing day. Now patterning technology can be easily completed without using a mask, which is a great help to the field of nanomanufacturing. The large-scale development of alternative nanolithography technologies, such as microcontact printing, nanoimprint lithography, and scanning probe lithography, is of great help to today’s industry. Due to their ease of use and operation, these methods are now used in almost all types of production. Although nanolithography was a very niche branch before, it has developed in areas such as maskless patterning, reduced equipment usage, reduced power consumption, reduced skilled workers, and reduced experimental settings, etc. The nanolithography becomes the first choice for the IC manufacturing industry and nanoelectromechanical systems (NEMS) [105].

### 2.4. Superplastic Nano Die Casting Technology

Liu et al. [106] invented die casting technology for crystalline metals. This technology is classified as a template method, which can directly mold metal into nanostructures at a temperature far below the melting point.

**Figure 6:**
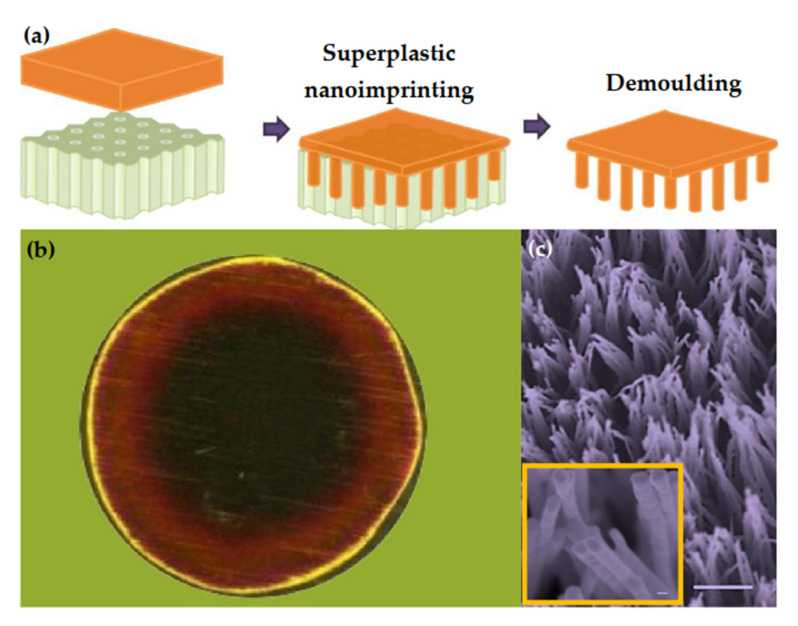
Flow chart of superplastic nano die casting technology: (**a**) Schematic of the SPNI process. (**b**) Optical micrograph of an as-thermoplastic-formed Au/Al_2_O_3_ template combination, which was prepared by SPNI under an applied force of 5 kN and holding for ∼60 min (scale bar, 1 mm). (**c**) microstructure of the surface in (**b**) observed by an electron microscope. The uniform rust-red color of the sample suggests that Au nanowire arrays have been replicated, which is verified by characterizing the sample under SEM after dissolving the Al_2_O_3_ template in KOH solution (**c**) scale bar, 5 μm. Inset figure (scale bar, 30 nm). Adapted from [106], under the terms of the Creative Commons CC BY license.

Figure 6a shows a schematic diagram of the preparation of crystalline metal nanostructures by die casting. First, a piece of metal is placed on a mold with nanostructures. The commonly-used mold is an anodic aluminum oxide (AAO) template. The second step is to heat the metal/template. When it reaches a specific temperature (generally the target temperature is set to 0.5 T_m_ < T < T_m_), pressure is applied to the metal and the template. At this time, the metal is plastically deformed and squeezed into the pores of the template. Generally, after superplastic nanomolding, chemical etching is used to remove the template, leaving an array of nanopillars grown on the surface of the metal substrate.

The superplastic nano-molding technology can prepare large-scale metal nano-pillar arrays, as shown in Figure 6b,c. Figure 6b shows the optical micrograph of Au flakes/AAO samples prepared by Au. The center of the sample shows a dark red color, which indicates that the surface of the sample is covered with microstructures. Figure 6c is the microstructure of the surface in Figure 6b observed by an electron microscope. The SEM image proves that the superplastic nanomolding technology has successfully replicated the pattern of the AAO template and prepared a nanopillar array with good uniformity.

**Figure 7:**
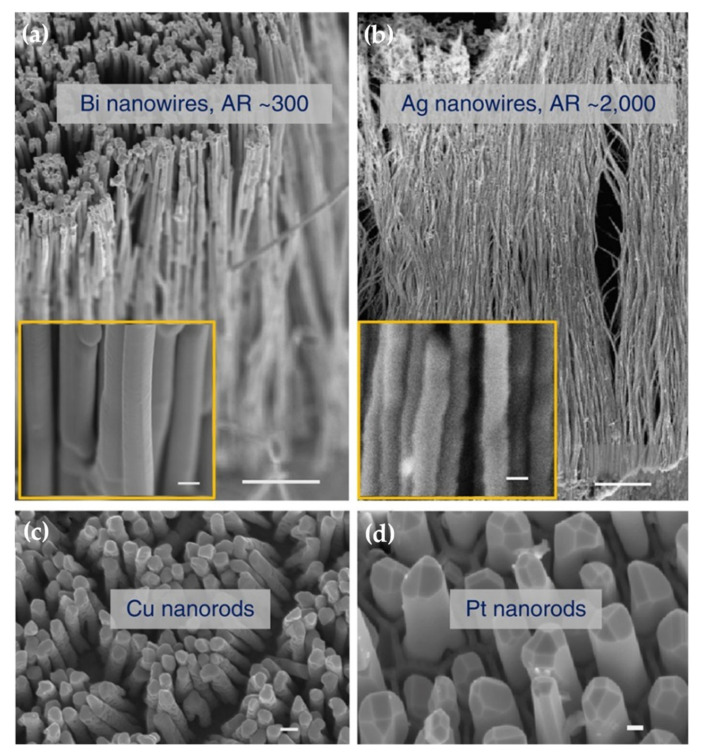
Nanostructure of Bi, Ag, Cu, and Pt metals: (**a**) SPNI, a piece of Bi by using a 200-nm Al_2_O_3_ template at 260 °C (closing to its melting temperature, Tm ∼273 °C) and under a force of 8 kN. Bulk Bi completely filled the Al_2_O_3_ template within 36 s, corresponding to an aspect ratio of ∼300 since the thickness of the Al_2_O_3_ template is ∼60 μm. Scale bar, 5 μm. Inset figure scale bar, 200 nm. (**b**) An extremely high-aspect ratio of ∼2000 for Ag nanowires was also obtained by SPNI of a piece of Ag into a 25-nm Al_2_O_3_ template at ∼700 °C, under an applied force of 15 kN and holding for 90 min. Scale bar, 2 μm. Inset figure scale bar, 25 nm. (**c**,**d**) Cu and Pt nanowire arrays were fabricated by SPNI at ∼550 and ∼820 °C, respectively. Scale bars, 200 and 100 nm, respectively. Adapted from [106], under the terms of the Creative Commons CC BY license.

**Figure 8:**
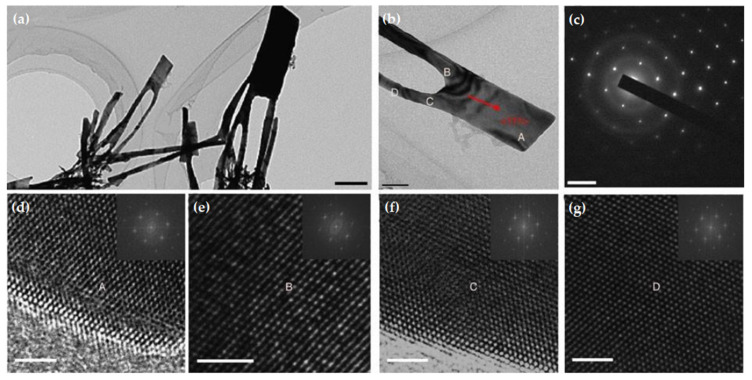
TEM analysis of Au nanostructures: (**a**,**b**) Topography images of prepared Au nanostructures. Scale bars, 200 and 50 nm, respectively. (**c**) Diffraction pattern of the Au hierarchical nanostructure in (**b**) showing a face-centered cubic single crystal structure and the axis of the nanostructure is determined along <111> crystallographic orientation. Scale bar, 5 nm^−1^. (**d**–**g**) High-resolution TEM images at the regions denoted by A, B, C, and D in (**b**) and fast Fourier transformations (insets) confirming the perfect single crystal of the Au hierarchical nanostructure. Scale bars, 2 nm. Adapted from [106], under the terms of the Creative Commons CC BY license.

The superplastic nano-molding technology is not only applicable to metal Au, but also applicable to metals Bi, Ag, Cu, and Pt. Figure 7 shows the Bi, Ag, Cu, and Pt nanostructures prepared by superplastic nanomolding technology. The nanopillar structure prepared by superplastic nanomolding technology has a perfect crystal structure. For example, Liu et al. [106] conducted TEM analysis on the Au nanopillars, and the results showed that the nanopillars prepared by superplastic nanomolding technology had a single crystal structure, as shown in Figure 8.

Figure 8a is a bright field image of the prepared nanostructures, and Figure 8c is a diffraction analysis of the nanopillar structure, which proves that the prepared nanopillars own a perfect single crystal structure. Figure 8d–g are the high-resolution TEM images and Fourier transform at positions A–D in Figure 8b, respectively, which once again prove that the entire nanopillar structure is a single crystal.

The superplastic nano-molding technology of crystalline metal at a temperature far below the melting point can prepare metal nanopillars with an aspect ratio of up to ~2000. The prepared nanopillars show a single crystal structure. At present, this technology helps to successfully prepare nanopillars with a diameter of only 5 nm, which is much smaller than the size range of metal grains. The preliminary verification has been carried out that this mechanism of plastic deformation may be derived from the creep mechanism controlled by lattice diffusion. Although the superplastic nano-molding technology has solved the problem of crystalline metal nano-preparation, the mechanism still needs to be further clarified. In addition, this technology relies significantly on the template, and the prepared nanostructures are usually columnar. Therefore, this technology still has certain challenges in the preparation of shape control.

### 2.5. Plasma Synthesis Method

At present, the most commonly used synthesis methods for metal oxide nanomaterials include the template method [107], hydrothermal or solvothermal synthesis [108], laser ablation [109], and thermal oxidation [110], etc. The template method can ensure good control of the morphology of the nanostructures, but is expensive and time-consuming [111]. Laser ablation synthesis can provide more controls on the morphology and quality of nanomaterials, but the process itself is expensive and not suitable for mass production [112]. Hydrothermal synthesis is fast, effective, and relatively inexpensive, but the morphology of the product is difficult to control only by the surfactants used [111]. The thermal oxidation method can well control the morphology of nanomaterials, but the repeatability of the preparation process is insufficient [113,114,115,116]. The metal substrate is treated with oxygen or other gases under plasma [117,118]. Compared with the thermal method, this method shortens the synthesis time by an order of magnitude. In order to form oxides through the thermal oxidation synthesis process, oxygen molecules must be adsorbed on the heated substrate and then decomposed into atomic oxygen, which then reacts with the metal [119]. On the contrary, the plasma already contains atomic oxygen species. Therefore, by using oxygen plasma as the treatment agent, the oxygen dissociation step can be skipped [120]. The plasma method is cheap, easy to scale, and environmentally friendly, which makes it very suitable for large-scale and even the industrial synthesis of nanostructures.

The reaction process of the direct current (DC) arc plasma method is a classic physical gas phase method. The DC arc plasma is a kind of thermal plasma. Energy is applied by direct current, and the applied energy is transferred by collision of electrons with neutral substances. The DC arc in the experiment is usually generated at a higher current and a lower voltage. After energization, the applied electrical energy quickly ionizes the gas in the reaction system into plasma, and the generated plasma is thermal plasma, which makes the center temperature of the arc several thousand kelvins. At high temperatures, the raw material target is quickly evaporated into atoms or ionized into ions. When these particles leave the central area of the arc, the reaction temperature drops sharply so that the particles recombine to form a stable crystal structure. The surrounding inert gas atoms or reactive gases collide violently to cause quenching or chemical reactions. In this method, the heat provided by the high temperature generated by the plasma is one reason for the evaporation of the raw material target, and the other main reason is caused by the action of the plasma itself.

Further studies have shown that [121,122] in addition to the formation of metal nanoparticles by direct thermal evaporation caused by the heating of the metal, it is more importantly caused by the action of different reactive gas plasmas, such as hydrogen. This action increases the synthesis speed of nanoparticles by ten times or even dozens of times. Someone proposed the mechanism of nanoparticle formation under hydrogen plasma conditions and believed that it was brought out by the escape of hydrogen from the alloy [121]. Five steps can be used to illustrate this. (1) Hydrogen atoms or ions enter the molten target. After the arc is triggered, hydrogen gas is decomposed into hydrogen atoms or ions. These active hydrogen atoms and hydrogen ions create a plasma environment for the entire reaction system. Since hydrogen atoms and hydrogen ions are a smaller size than hydrogen molecules, it is easier to enter the molten raw material target, which is theoretically estimated to be 10^5^–10^8^ times that in the gaseous state. (2) The formation of molecular hydrogen. When hydrogen atoms or ions enter the molten target, the temperature quickly drops to about 1500 °C, and the solid solution of hydrogen atoms becomes over-saturated, thereby combining into hydrogen molecules. (3) The formation of bubbles. When the concentration of hydrogen molecules further increases to reach saturation, hydrogen bubbles will be formed. (4) The formation of target vapor. When hydrogen molecular bubbles are formed, the target atoms will also evaporate into the bubbles to form target vapor. Due to the large evaporation area and low pressure at this time a large amount of target vapor will form. (5) The escape of bubbles. When the bubble grows to a certain extent, it will leave the molten target and carry the raw material particle vapor out. The carried-out particles will nucleate and generate nanoparticles after leaving the central area [123]. Therefore, changing the proportion of hydrogen in the experimental gas will affect the evaporation rate of the raw materials, and will affect the size and morphology of the generated nanoparticles.

**Figure 9:**
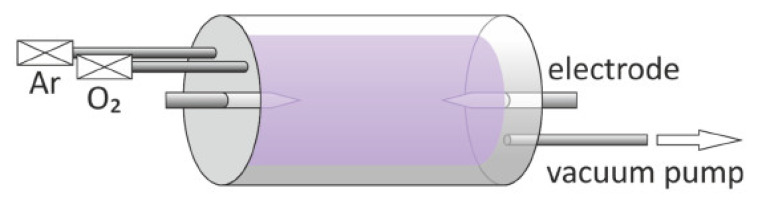
Schematic diagram of a custom-made plasma-enhanced horizontal tube furnace deposition system. The vacuum-sealed glass cylinder reactor has two electrodes with pointed ends from each side, and these two electrodes are used to ignite plasma. Adapted from [124], under the terms of the Creative Commons CC BY license.

**Figure 10:**
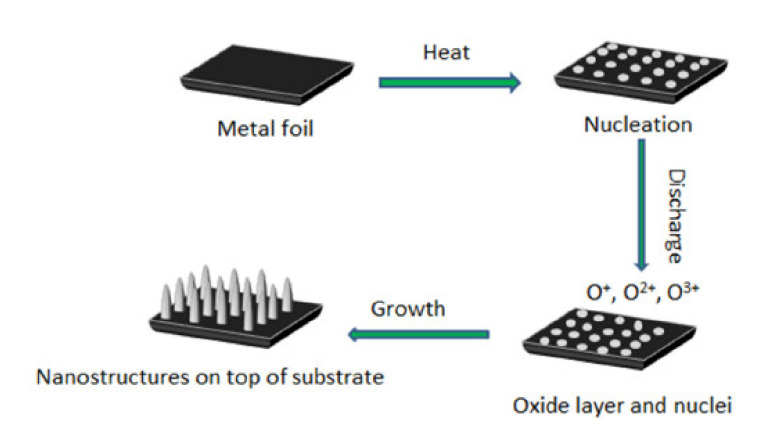
Schematic illustration of the initial stages of the growth process for the metal oxide nanostructures produced under plasma. These stages lead to the nucleation, nanowire growth, and formation of a thin oxide layer. Adapted from [124], under the terms of the Creative Commons CC BY license.

As shown in Figure 9 and Figure 10, Guo et al. developed and described a customized plasma-enhanced horizontal tube furnace deposition system, namely plasma-enhanced thermal oxidation (PETO), to synthesize a variety of metal oxide nanostructures, including ZnO, Fe_2_O_3_, CuO, etc. Using the PETO method, stable and high-quality metal oxide nanomaterials can be produced at a lower processing temperature and in a shorter growth time [124].

### 2.6. Other Preparation Methods

Self-assembly is a method of preparing nanostructures. This method does not rely on external forces, and combines the dispersed microstructures through the interaction force of the structure itself. This interaction force is generally Van der Waals force, hydrogen bond, etc.

In addition, in recent years, people have noticed that living microorganisms such as bacteria, fungi, and plants have great potential in the synthesis of metal nanoparticles like synthetic sulfide (CdS) [125], titanium/nickel, titanate [126], zirconia [127], gold [128], and silver [129], etc. Application of micro-organisms is an environmentally friendly and benign synthetic route, which provides a green technology for the control and preparation of nanostructures. For example, M. Gericke used the cultivation of two fungi to synthesize gold nanoparticles of different shapes and sizes in the cells. The size can be adjusted by controlling parameters such as pH, temperature, gold concentration, and exposure time. These methods allow the production of uniform metal nanopatterns. However, due to the time-consuming, multi-step process, the cost is high, and the preparation of different materials is also restricted.

## 3. Characterization Methods

For metal nanostructures, we usually need to use scanning electron microscope (SEM) to characterize the surface morphology, element composition, microstructure, etc., and use ultraviolet photoelectron spectroscopy (UPS), X-ray photoelectron spectroscopy (XPS), etc. to measure surface work function, energy level arrangement at the interface, and the chemical composition of the material. The following is a related introduction about them.

### 3.1. Scanning Electron Microscope (SEM)

Scanning electron microscopy is a technique in which electron beams act on the surface of a sample to obtain information and structure of the sample by collecting secondary electrons and backscattered electrons. When incident electrons collide with atomic electrons, they excite secondary electrons. By detecting the secondary electrons, the surface morphology of the sample can be obtained. When incident electrons collide with atomic nuclei, backscattered electrons are generated. The intensity of backscattered electrons increases with the increase of the atomic number. Therefore, backscattered electrons can be used to detect materials in the sample. In addition, when the electron beam acts on the sample surface, the composition of the sample can also be measured by the generated X-ray spectrum. The scanning electron microscope is composed of an electronic optical system, a scanning system, a signal detection amplification system, and an image display system [130]. At present, there are mainly low-voltage SEM, environmental SEM, analytical SEM, field emission SEM, etc. If the SEM is equipped with an X-ray energy spectrometer device, it can analyze the composition of the micro-area and observe the morphology of the microstructure at the same time. It is a scientific research instrument with a wide range of applications. At present, a major development trend of scanning electron microscopy is to conduct research on the technology of combining SEM with other equipment.

### 3.2. Scanning Tunneling Microscope Technology (STM)

One can operate and control single atoms through a scanning tunneling microscope (STM), which has played an important role in the research of nano-metal materials. STM is a kind of microscope with high resolution and has unique advantages. It can use tunneling current to study the surface morphology and surface electronic structure of nano-metal particles. STM can observe structural defects such as atomic hills, platforms, steps, and holes on the surface. STM can also observe and study the structure of atoms and electrons on the surface of nano-metal materials. It can also measure surface fluctuations by observing the microscopic three-dimensional images of the material. This technology satisfies people’s requirements to directly observe atoms. STM does not cause damage to the sample during imaging, and reduces environmental constraints when performing experiments. STM also has strong applicability and is an important tool for technicians to conduct research on nano-metal materials.

### 3.3. Atomic Force Microscope (AFM)

Although AFM has a similar working principle to STM, AFM has its own unique advantages. AFM has lower requirements for samples, and therefore has a wider application range. In the research of nano-metal materials, AFM can analyze the surface morphology and can be combined with other equipment such as STM, TEM, etc. to study nano-metal particles.

### 3.4. Transmission Electron Microscopy (TEM)

Transmission electron microscopy (TEM) is one of the main instruments for studying the microstructure of materials. It can observe and study the distribution, microscopic morphology, and crystal structure of nanoparticles. Researchers use transmission electron microscopy combined with energy spectrometer (EDS) and other technologies to simultaneously achieve high-efficiency characterization of the crystal structure of nanomaterials and the distribution of elements within the material. In addition, transmission electron microscopy can also accurately measure the particle size distribution and size of nano materials, and test nano particles inside metal materials with high accuracy. In addition, the application of in-situ analysis technology in the TEM system has made breakthrough progress. For example, by introducing a certain atmosphere, liquid phase, or raising to a certain temperature in the TEM, the changes of microscopic morphology and structure for the material can be observed as well as studied in real time and with high resolution.

### 3.5. X-ray Photoelectron Spectroscopy (XPS)

X-ray photoelectron spectroscopy (XPS) is a technique for analyzing the chemical properties of the surface of a substance. When a X-ray goes through the filter to the sample, the electrons are activated and ejected by the action of the ion gun. The energy distribution is processed by the electron energy analyzer. Finally, the accelerated electrons after passing through the electron multiplier enter the scanning and recording system for recording comparison, and the result is obtained. XPS is a surface analysis instrument for measuring surface element analysis, band structure and chemical state, and chemical state imaging.

### 3.6. Ultraviolet Photoelectron Spectroscopy (UPS)

Ultraviolet photoelectron spectroscopy is a technique that ultraviolet light irradiates the surface of the sample, causing photoelectrons to be emitted from the surface of the sample. These electrons are received by the energy analyzer, and the electron detector counts the number of electrons, and finally the UPS energy spectrum is shown on the display, which can be used to characterize the energy level structure and surface work function of the sample’s valence band [131].

Since nano-metal materials are only a few to tens of nanometers in size, the special effects and different types of defects caused by them require higher-resolution instruments for analysis. In the current research stage, various electron microscopy technologies have their own unique advantages and disadvantages. According to different influencing factors such as research content and experimental parameters, choosing the most appropriate electron microscopy technology is conducive to better developing nano metal materials. For example, the particle size of cluster materials in only a few atoms. AFM and STM can be used for combined analysis. Nanocrystalline structure materials can be studied by a combination of SEM and TEM.

## 4. Performance and Application

Nano-metal structured materials are composed of metal particles with a size ranging from a few nanometers to one hundred nanometers. Some phenomena of nano-structured materials can be understood according to the following three structural models [132].

1. Variable interface model: Nano-metal structure materials have many interfaces because the particles (crystal grains) are nano-scale, and the energies existing on the interfaces are very different. The energy can be affected by many aspects such as the interfacial atomic distance, arrangement, coordination number, etc. For nanostructured materials, the change of the lattice constant will change the surface translation period, and even destroy the surface translation period. Such complex surface states and interactions possibly lead to unique magnetic, electrical, and optical properties in nano-metal materials.
2. Interface defect model: The volume of nanoparticles is very small. When the interface composition changes, the order of the atomic arrangement at the grain boundary will also change, resulting in more defects in the interface. Structural defects will exert a great impact on the super-plasticity and strength of the material.
3. Gas-like model: When the atoms are arranged on the interface of the nano-metal structure material, they are disordered, e.g., in a gas state, and not arranged according to a certain rule. However, when professional researchers have gradually deepened the research on the microstructure of nanomaterials, they discovered that nanostructured materials are not in completely disordered states, but a combination of disorder and order.

### 4.1. Nano Metal Structure Material Characteristics

Although metal nanostructures have broad application prospects, studies have shown that the performance of metal nanostructures significantly depends on their geometric dimensions and shapes. This is mainly because the characteristic size of the material (the size of the internal microstructure or the geometric size of the nanomaterial) usually determines its performance (mechanical, electrical, optical, or magnetic, etc.).

Taking metal materials as an example, the internal crystal grain size has a great influence on the mechanical properties of the material. Generally, the yield strength of metals can be improved by refining the grain size, where the yield strength (σ_ys_) is inversely proportional to the square root of the average grain diameter (d) (σ_ys_ ∝ d^−1/2^). This relationship is usually applicable to the size from sub-millimeter to tens of nanometers grains.

More than 60 years ago, S. S. Brenner [133] observed a substantial increase of the yield strength (close to the theoretical limit) in the tensile test of a single crystal metal rod with diameter of 1 μm. In contrast, J.G. Sevillano [134] did not observe changes in strength in sub-micrometer diameter samples. These studies are only the beginning to exploring the difference between millimeter- and micrometer-sized rod materials.

**Figure 11:**
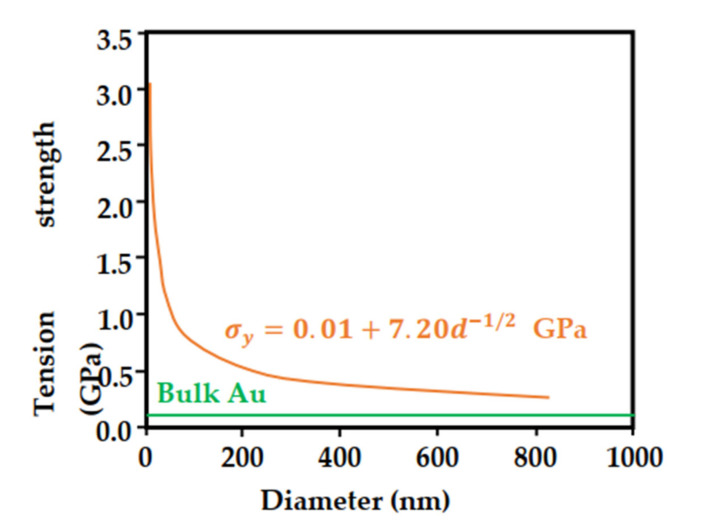
The relationship between the tensile strength of Au nanopillars and the diameter of nanopillars.

In the preparation process of nanostructures, the focused ion beam method is good for the preparation of one-dimensional nanopillar structures. Using this technology, people have prepared nanopillar structures of various sizes in different materials to study their mechanical properties. Taking Au nanopillars as an example, a large amount of experimental data show that the tensile strength of gold nanopillars is obviously size-dependent, and the smaller the diameter of the nanopillars, the higher the tensile strength (shown in Figure 11). This relationship can be expressed by the Hall–Petch formula [135]:

(2) $$\sigma_{y}=\sigma_{0}+kd^{-\frac{1}{2}}.$$

Among them, $\sigma_{y}$ and $\sigma_{0}$ are the yield stress and internal friction, k is the Hall–Petch constant, and d is the size of the grain, which can generally be understood as the characteristic size.

The Hall–Petch formula was originally used to describe the relationship between the yield point and ferrite grain size in low-carbon steel, and it was gradually summarized as “the smaller the size, the higher the strength”, and the meaning of d is also extended from the intrinsic grain size to the geometric size of the sample, the thickness of the film and the layer spacing of the hierarchical structure [136,137,138], etc.

Using the Hall–Petch formula to fit the tensile strength of Au nanopillars with different diameters, the empirical formula for the tensile strength and diameter of Au nanopillars can be obtained [138]:

(3) $$\sigma_{ys}=0.01+7.2d^{-\frac{1}{2}}GPa.$$

Michael [139] studied the influence of the grain size of the metal sample Ni_3_Al-Ta on strength and plasticity. Compression tests were performed on the cylindrical structures with diameters of 0.5–20 μm. For comparison, they refer to the compression experiment of bulk materials and observe the failure modes of samples with different diameters.

**Figure 12:**
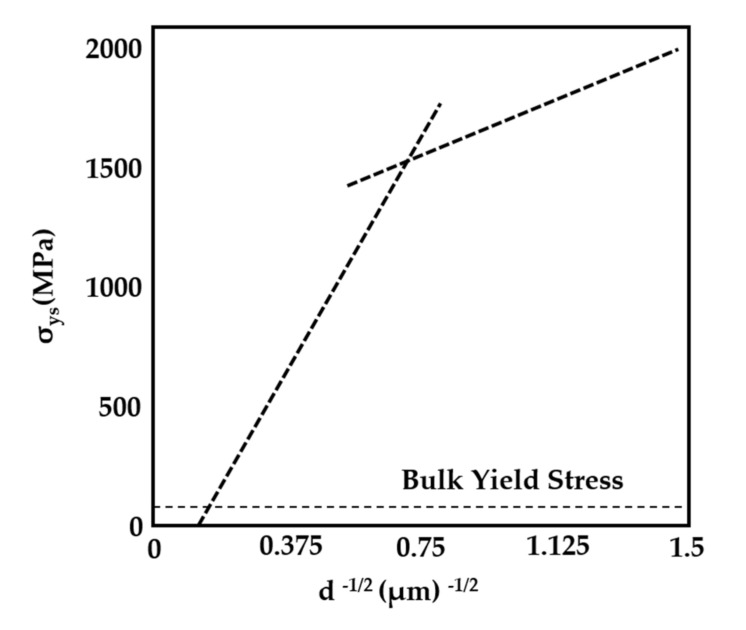
The relationship between the yield strength of Ni_3_A1-Ta and d^−1/2^.

In addition, Michael calculated the relationship between the yield strength of Ni_3_A1-Ta and the sample diameter (Figure 12). The results show that the yield strength of Ni_3_A1-Ta is proportional to the reciprocal of the square root of the sample diameter, which is similar to the grain size effect described by the Hall–Petch formula.

The change of nanostructure has great impact on the strength, plastic deformation ability, tensile performance, and other mechanical indicators of metal materials. Take gradient structure materials as an example, gradient materials are materials whose grain size gradually increases or decreases in a certain direction. Compared with ordinary materials, gradient materials have ultra-high strength and hardness, excellent stretching plasticity, etc.

For example, Lin [140] studied the influence of grain gradient on the strength of metallic Ni by preparing metallic Ni with different gradient structures. In the experiment, it was ensured that the coarse and fine grains of each sample had the same size, respectively. The only variable was the speed of the gradient from coarse to fine grains.

The speed of the grain size change in the gradient material has a greater impact on the strength of the metal. Generally speaking, the structure strength of the gradient material whose grain size changes uniformly is greater.

### 4.2. Metal Nanostructure Performance and Application

The essential difference between nanostructured materials and macroscopic bulk materials is that nanomaterials have a larger specific surface area, which brings different macroscopic and microscopic properties of the same material. Many studies have shown that the properties of nanomaterials are closely related to the geometric characteristics of nanostructures. By changing the morphological characteristics of nanometals, nanometal materials can play an important role in different fields (mechanics, electricity, optics, etc.) [141,142,143,144].

#### 4.2.1. Nanosheet Photothermal Therapy

Due to its photothermal stability and biocompatibility in the near infrared (NIR) region [145], nanostructured metals may become the main candidate materials for photothermal therapy. Photothermal therapy, also known as thermal ablation and optical hyperthermia, refers to a treatment method that uses the photothermal effect of a photothermal conversion agent under laser irradiation to generate a local high temperature in order to cause the death of cancer cells [146]. Photothermal therapy is an effective method to process tumors and bacteria in recent years. The principle is using materials to enter the human body [147,148] and actively or passively stay inside or near the tumor tissue, convert external light energy into heat energy, and increase the temperature of the tumor area to a temperature that can kill the cells [149], that is, the tolerance temperature of tumor cells and bacteria is 42 °C. The photothermal effect only occurs directly around the photothermal conversion agent, and the local temperature may be from tens to hundreds of degrees higher than the physiological temperature (in a short period of time). This means that photothermal heating can target tumors rather than healthy tissues, minimize the side effects of cancer therapy, and act as an efficient, minimally invasive, and highly specific treatment method. An ideal photothermal reagent should meet the following requirements: Good biocompatibility, good light stability, strong absorption in the near-infrared region, high photothermal conversion efficiency, easy modification of the particle surface, and effective accumulation in tumors.

So far, many types of photothermal nanomaterials have been extensively developed, including graphene [150,151], metal iron nanoparticles [152], cobalt-based nano-flakes [153] and gold nanoparticles [154], copper chalcogenides (such as CuS and Cu_2-x_ Se), and other nanoparticles [155,156]. However, these nanomaterials have certain shortcomings in biological and medical applications. They are composed of heavy metals such as iron, cobalt, gold, copper, tungsten, etc. At the same time, they have a certain degree of toxicity, that can cause serious side effects after being injected into the body [157]. Many in vitro and in vivo studies have shown that these nanocomposite materials are cytotoxic because they oxidize the heavy metal elements that make up the nanomaterials into free radicals and release them into the blood through different biochemical pathways. At the same time, if heavy metals accumulate inside the cell, it will cause cell death in severe cases. In addition, the synthesis part of nanocomposites also has limitations. They require expensive and toxic chemicals as raw materials, the process steps are complicated, time-consuming and material-consuming, and the principle is complicated. Therefore, to further improve the effect of photothermal therapy, it is also necessary to develop new photothermal preparations with good biological safety and simple synthesis.

In the past few years, many nanosheets have been used in photothermal therapy. As a result of the layered structure and large surface area, they have higher drug loading, more surfactant groups, and higher photothermal conversion efficiency than ordinary nanoparticles. An ideal photothermal agent should have a sufficiently high mass extinction coefficient to ensure excellent near-infrared spectrum- (NIR) induced photothermal performance, and also meet the complex biocompatibility requirements in the physiological environment. For example, the (Pd) nanosheets in an aqueous solution are irradiated with a near-infrared laser, and the near-infrared photothermal efficiency is monitored at different temperatures. It was found that after 10 min of near-infrared laser irradiation, when the solution contains nanosheets, the temperature was observed to rise from 28 °C to 48.7 °C. In contrast, the 1-mL aqueous solution of nanosheets only increased by 0.5 °C under near-infrared laser irradiation. Applying this technique to liver cancer cells, almost 100% of the cancer cells are killed within 5 min of laser irradiation, and the effect is remarkable. After 30 min of near-infrared laser irradiation, the photothermal stability of Pd nanosheets is better than that of Au and Ag nanosheets. In addition to the nanosheets, gold nanoprisms exhibit excellent photo-thermal stability under a longer near-infrared laser (wavelength 1064 nm) irradiation time. This is because the infrared light has a higher tissue penetration ability and lower blood and light absorption properties of soft tissues [158].

**Figure 13:**
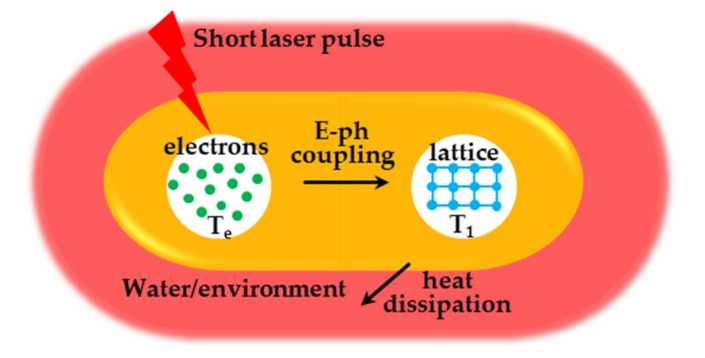
The physical process of photothermal conversion of gold nanorods activated by femtosecond laser pulses.

The strong light absorption capacity and non-radiative energy dissipation characteristics make it possible for the metal nanostructures to be used for photothermal therapy, such as gold nanoparticles. Link et al. [159] used femtosecond transient absorption spectroscopy to study the photothermal heating process in gold nanoparticles. The near-infrared laser pulse absorbed by the gold nanoparticles excites the free electrons in the plasmon band, thereby generating thermionic pulses. The hot electron pulse is rapidly cooled by the interaction of electrons and phonons, and collides with the gold lattice, heating it to thousands of degrees in about 1 ps (depending on the laser power). Then, heat is transferred from the nanoparticle to its surrounding area on a time scale of about 100 ps through the phonon-phonon interaction, causing the temperature of the surrounding medium to rise by tens of degrees. The schematic diagram of the photothermal effect in gold nanorods is shown in Figure 13.

**Figure 14:**
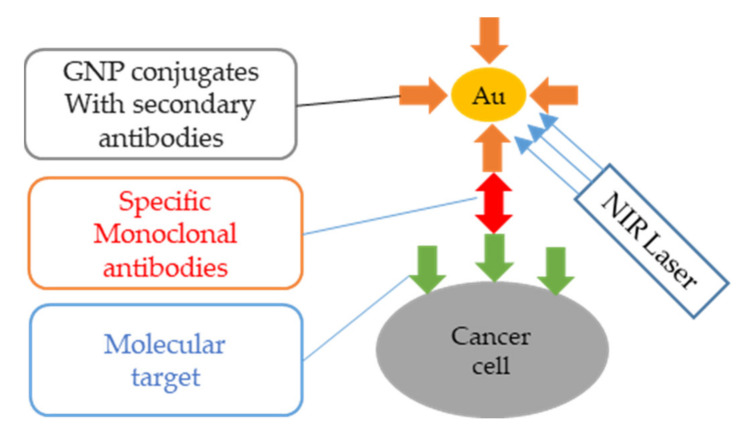
Schematic diagram of photothermal treatment scheme using AuNPs to actively deliver to cancer cells.

Researchers target the characteristics of tumors such as certain protein receptors on the surface of cancer cells (such as folate receptors) or the special internal environment of the tumor (such as hypoxia) to modify nanoparticles to increase their enrichment in the tumor, thereby improving the photothermal therapy effect. Figure 14 is a schematic diagram of the application of Au nanoparticles (AuNPs) in tumor photothermal therapy. The recommended name for this method is plasmonic photothermal therapy (PPTT). In addition, AuNPs-antibody conjugates can be used for diagnosis and plasma photothermal therapy, the so-called diagnostic method.

**Figure 15:**
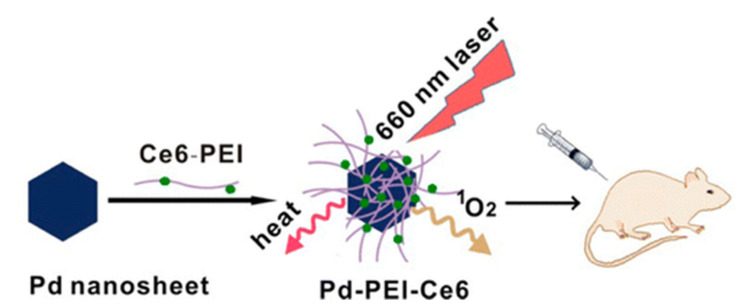
Palladium (Pd) nanosheet composite materials used in photothermal therapy. Reprinted with permission from Ref. [160]. Copyright 2014 American Chemical Society.

Zhao et al. [160] successfully used Pd nanocomposites in mice to combine near-infrared photodynamics and photothermal treatment for cancer, and this multifunctional treatment system has important prospects in the development of a new generation of cancer treatment systems, as shown in Figure 15.

#### 4.2.2. Surface Enhanced Raman Scattering (SERS) with Porous, Core-Shell Structure

Metal nanostructures have local electromagnetic field enhancement capabilities and are widely used as the substrate for SERS. In 1928, Indian physicist Chandrasekhara Raman firstly discovered that when a beam of light passes through a transparent medium, most of the light will be transmitted through the substance, absorbed by the substance, or reflected on the surface of the substance. A part of the light will be scattered on the surface of the medium molecules and the frequency of the scattered light will change. The scattered light with frequency changes is called Raman scattering. Raman scattering, also known as the Raman effect, is a phenomenon in which the Raman scattering signal of molecules adsorbed on a specially prepared substrate is greatly enhanced. In the incident light, the intensity of Raman scattering is about 10^–3^ than that of Rayleigh scattering. Due to the cross-sectional area of Raman scattering being very small and the Raman signal being very weak, the incident light intensity is only about 10^–3^, coupled with the combined factors such as fluorescence interference and backward laser technology, resulting in the limited early application of Raman technology. In 1974, Fleishmen [161] discovered that the pyridine molecule was adsorbed on the surface rough of the Ag electrode, a strong Raman signal was generated; from 1977 to 1979, the two research groups of Van Duyne and Creighton independently summarized the phenomenon experimentally and theoretically, and the results showed that it was a regular phenomenon based on the rough surface. It is denoted as the surface-enhanced Raman scattering effect. In addition, Moskovits et al. [162] have also verified through experiments that surface plasmons are the main reason for the enhanced Raman signal generated by the rough electrode.

SERS is Raman scattering amplified by molecules adsorbed on the metal surface. It is a highly sensitive, highly selective, and fast response surface detection technology. It can achieve ultra-sensitive detection down to the single-molecule level through the nanostructures of precious metals (such as gold, silver, and copper), and is used to detect a variety of molecules [163,164]. It has unique advantages in non-destructive testing in the fields of analytical chemistry, food safety, and environmental monitoring.

Metal plasmon nanostructures, such as gold, silver, and other precious metal nanoparticles are widely used in the field of photoelectric sensing due to their unique physical and chemical properties. As a non-invasive optical sensing technology, SERS detection is widely used in the fields of catalytic reaction monitoring [165], food safety [166], bacteria detection [167], environmental detection [168], and pesticide detection [169], etc. In SERS detection, in order to obtain stronger signals, researchers have designed SERS substrates with different morphologies of nanostructures by precious metals. Plasma nanostructures with sharp corners are very effective in maintaining SERS activity. SERS activity can increase the electromagnetic field (E-field) and produce “hot spot” effects [170].

**Figure 16:**
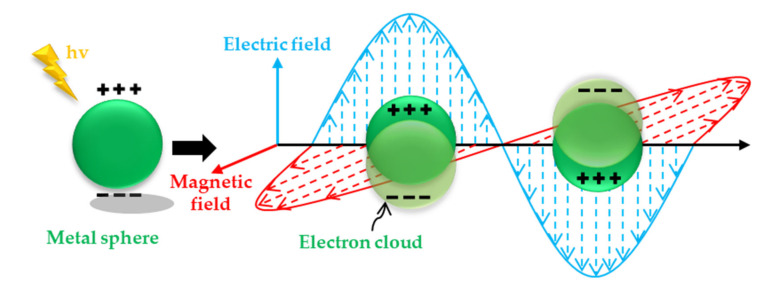
Schematic diagram of a localized surface plasmon.

It has been many years since people first discovered the SERS effect. Due to the complexity of the SERS effect, the enhancement mechanism has not yet been determined. There are two main unified views [171], the physical enhancement model and the chemical enhancement model. The physical enhancement model is that the enhancement of the Raman signal is realized by the electromagnetic field generated by the local surface plasmon (LSPR) activated on the rough surface of the metal nanomaterial [164], so this physical enhancement model is also called electromagnetic field enhancement (localized surface plasmon resonance of metal nanoparticles). Electromagnetic field enhancement dominates the contribution of SERS [172]. Theoretical calculations show that [173] the maximum enhancement factor (EF) achievable by electromagnetics is about 10^11^, and the enhancement factor based on the chemical mechanism is about 10–10^4^. The SERS substrate is used as the carrier of the molecule to be detected, and its activity and stability performance is the key to affecting Raman signal amplification and spectral quality. Specifically, when light is incident on the interface between the metal nanostructure and medium, the surface of the metal nanostructure will oscillate collectively. When the free electrons on the surface are coupled with electromagnetic waves, it will form a near-propagation along the surface of the metal nanostructure. This phenomenon in which the metal substrate causes surface plasmon resonance under the action of excitation light is called LSPR. When a certain wavelength of incident light irradiates the surface of the metal substrate, the free electrons on the metal surface produce collective coupling oscillations, so that the electromagnetic field on the metal surface is locally enhanced. The scattering cross section of the molecule to be measured in the local electromagnetic field is greatly increased, and the Raman of the molecule is greatly enhanced, and the enhanced factor (EF) can be as higher as 10^6^–10^9^ times. The enhancement effect of this electromagnetic field is closely related to the particle size, structure, and type of the precious metal substrate. Figure 16 is a schematic diagram of the LSPR of spherical metal nanoparticles. If this electromagnetic field is restricted to a small range on the metal surface, it is called a localized surface plasmon. At this time, if the probe molecule is in this area, its Raman signal has been significantly enhanced. When the surface of the metal nanomaterial has high roughness, a large number of hot spots will be formed. In the slit area or the area with a larger curvature, the electromagnetic field can be amplified to form more surface plasmons, so it has high surface roughness [174]. The metal nanomaterials are ideal SERS substrates. The current common SERS substrates roughly include metal electrode active substrates, metal sol active substrates, solid substrates with self-assembled nanoparticles, and directly processed solid substrates.

**Figure 17:**
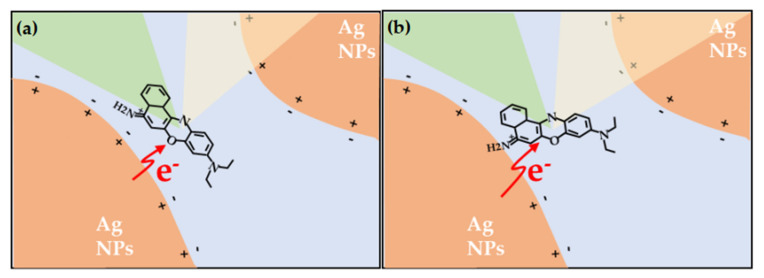
The charge transfer mechanism of single-molecule electrochemistry: (**a**) Before and (**b**) after.

The chemical enhancement model (the charge transfer between the molecule to be tested and the surface of the metal nanoparticle), which is different from physical enhancement, is a short-range enhancement that only targets certain specific molecules. In the chemical enhancement theory, the generally accepted mechanism of charge transfer, the theoretical model believes that when molecules are adsorbed on the surface of the precious metal substrate, the molecules to be tested and the metal substrate contact to form a chemical bond, and the Fermi energy level of the metal substrate is located at the highest occupied orbital (LUMO) and the lowest unoccupied orbital (HOMO). Under suitable incident light excitation, electrons at the Fermi level are light-activated to transition to the LUMO level of the molecule to be measured, or electrons at the HOMO level of the adsorbed molecule transition to the Fermi level of the metal substrate. These charge transfer processes increase the effective polarizability of the molecule and enhance the Raman signal [175]. The enhancement coefficient of the chemical enhancement model is lower than that of the physical enhancement model, generally 10^2^. Figure 17 is a schematic diagram of the electrochemical charge transfer mechanism of a single molecule. Combining the two enhancement effects introduced above, it is found that the Raman signal intensity of the tested molecule is not only related to the local electromagnetic field intensity generated on the surface of the metal substrate structure, but also related to the polarizability of the molecular system.

**Figure 18:**
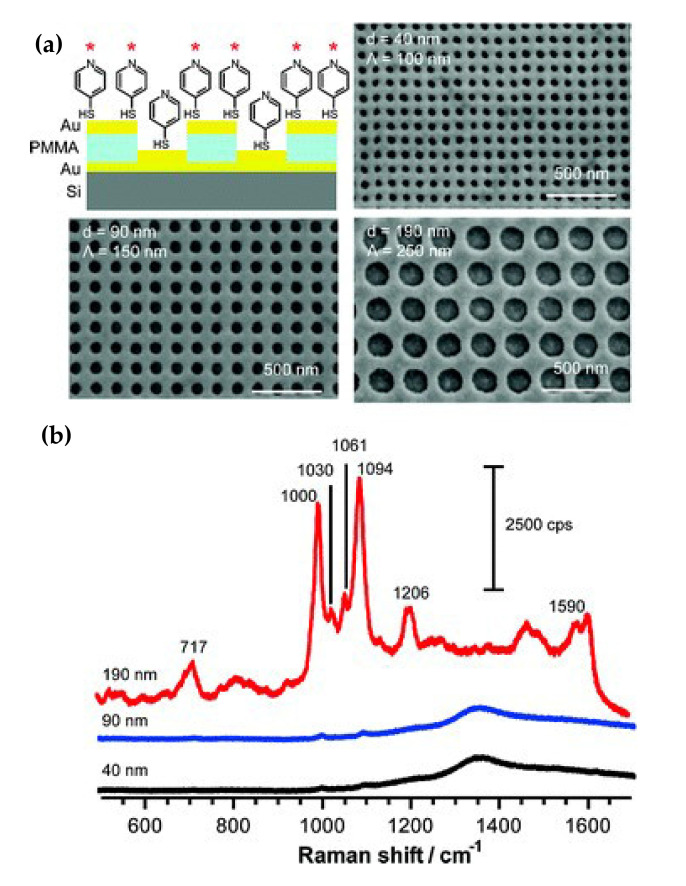
Surface-enhanced Raman scattering of gold nanohole array: (**a**) Schematic of side view of gold thin film with nanoholes and a physically separated layer of isolated gold disks at the bottom of the well. 4-MP molecules adsorbed on the surface with an asterisk to illustrate where the local electromagnetic field is enhanced. SEM images of three gold nanohole arrays with diameters of 40, 90, and 190 nm and the gratings of 100, 150, and 250 nm, respectively. The thickness of gold films is 50 nm. The depth of wells is ∼100 nm measured by tapping mode AFM. (**b**) SERS spectra of 4-MP adsorbed on three gold nanohole arrays with different diameters activated by a 785-nm laser. Reprinted with permission from Ref. [163]. Copyright 2008 American Chemical Society.

Yu et al. [163] studied the use of electron beam lithography to make gold nanoholes and nanodiscs arrays with precisely controlled size and spacing, and tested the surface-enhanced Raman scattering, as shown in Figure 18. These nanostructures exhibited a strong SERS signal when activated at 785 nm, while the SERS signal was weak at 514 nm. The results show that with the increase of the nano-aperture, the enhancement of the local surface plasmon resonance wavelength shifts to the near-infrared direction.

In addition, Huang et al. [176] described the importance of the sharpness of metal nanostructures in SERS. By controlling the acute angle of the nanostructure, the SERS signal can be regulated, but the stability of the acute angle is a challenging issue. In order to overcome this problem, they synthesized the Pd–Ag core-shell and other bimetallic nanostructures. This Pd–Ag core-shell structure owns good stability and shows relatively stable signals in SERS applications.

#### 4.2.3. Surface-Enhanced Fluorescence (SEF) of Nanoparticles

Fluorescence technology has a wide range of applications in microscopic imaging, optical devices, medical diagnosis, and other fields. Scientists have shown great interest in improving the fluorescence sensitivity of fluorescent carriers. Due to the needs of many potential applications, fluorescence sensitivity of a single molecule has become a challenging topic [177,178]. For this reason, surface-enhanced fluorescence (SEF) technology was developed. The SEF effect means that when a fluorescent species is close to the metal nanostructured substrate, its radiation behavior will be regulated. Under appropriate conditions, the spectral radiation intensity of fluorescent species will increase compared to the intensity in free state [179]. SEF is highly dependent on the near-field coupling between the activated state fluorophore and the surface of the object. In particular, nanostructured surfaces with localized surface plasmons are effective SEF substrates. Due to the interaction between molecules and nanoparticles, the distance between molecules and a plasma nanostructure has a greater impact on the SEF effect [180,181]. Generally speaking, there are three factors that affect surface-enhanced fluorescence: (1) The substrate metal generates surface plasmon resonance under the excitation of an external light field to cause local field enhancement; (2) the increases in the quantum yield and radiation rate of fluorescent species; and (3) energy transfer between the substrate metal and fluorescent species. The specific explanation is as follows: When the incident light frequency of the external light field is coupled and matched with the natural frequency of the substrate metal and the emission band of the fluorescent species, a collective electronic oscillation mode will be formed on the metal surface and the local field will be enhanced. In the case of the enhancement of the local field, the number of free electron transitions of the fluorescent species, the fluorescence radiation rate and quantum yield increase. However, if the distance between the substrate metal and fluorescent species is too close, the activated fluorescent species will transfer energy to the substrate metal in a non-radiative manner, and the substrate metal after absorbing the energy will produce a thermal effect and cause the radiation efficiency of fluorescent species to decrease. The phenomenon of fluorescence quenching occurs. In the study of surface-enhanced fluorescence effects, fluorescence enhancement and fluorescence quenching are mutually restrained processes, which is mainly related to the morphology and the size of metal nanostructure surface and the distance between fluorescent species and a metal surface.

In early studies on enhanced spectral effects, electrochemical polishing or physical polishing methods are usually used to prepare metal nanostructured substrates. Although the prepared substrate has poor reproducibility and other shortcomings, as a traditional method for preparing a reinforced substrate, it has a simple preparation process and can obtain a metal substrate with a micro-nanostructure. It has important research value in the study of surface-enhanced fluorescence effect [182]. Studies have shown that metal nanoparticles will produce LSPR under specific excitation conditions [179], and a strong local electric field will be formed around them. Under the action of a local electric field, the number of free electrons in high-energy states in fluorescent species increases and their transition frequency is accelerated, and the spontaneous emission of fluorescent molecules is enhanced. In 1982, Weitz et al. [183,184] used silver island film (SIF) as a substrate to study the fluorescence radiation characteristics of Eu^3+^ ions. Experiments have found that under the action of laser pulses, Eu^3+^ fluorescent radiation using SIF as a substrate is enhanced. The author made a quantitative analysis of his experimental phenomena based on quantum yield, and believed that quantum yield played a decisive role in the fluorescence radiation of Eu^3+^, which laid an experimental and theoretical basis for the study of surface-enhanced fluorescence effects.

Metal nanoparticles are often used in the study of surface-enhanced fluorescence effects. The preparation method of the substrate is simple and time-consuming. Under proper conditions, the substrate exhibits a good fluorescence enhancement effect. Zhu et al. [183] used Au nanoparticle (AuNP) as a substrate and found the spectral enhancement of the Rhodamine B fluorescent molecule under the excitation of an external light field. In addition, the main feature of Au nanorod (AuNR) is that it has two plasmon resonance absorption peaks. By changing the aspect ratio of AuNR, the position of the plasmon absorption peak can be changed, and the perfect match between the nanorod and emission peak of the fluorescent species can be achieved which can enhance the fluorescence signal intensity of the luminescent center. Zhu et al. [183] achieved effective regulation of the peak-to-peak value of the longitudinal plasmon absorption by adjusting the aspect ratio of AuNR. Disordered precious metal nano-sol particles with special optical properties prepared by wet chemical methods can be used as metal nano-antennas to regulate the fluorescent radiation of luminescent center, and have been successfully applied in the fields of biosensing [185] and substance detection [186], etc.

**Figure 19:**
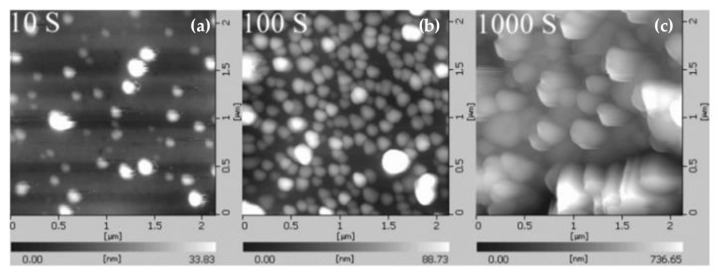
Silver nanostructures formed by electrostatic potential deposition at different times. Reprinted with permission from Ref. [178]. Copyright 2007 American Chemical Society.

**Figure 20:**
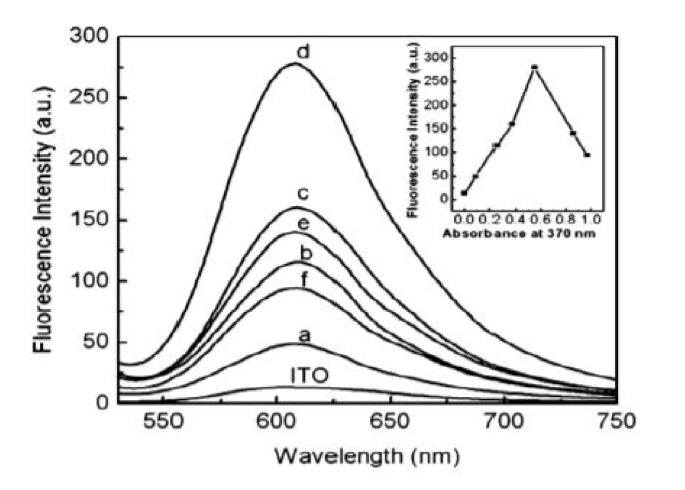
Fluorescence spectra under different electrodeposition times: (a) 10 s; (b) 50 s; (c) 100 s; (d) 200 s; (e) 500 s; and (f) 1000 s. Reprinted with permission from Ref. [178]. Copyright 2007 American Chemical Society.

Shang et al. [178] introduced a fast and simple method, electrostatic potential deposition technology, to enhance fluorescence by depositing metal particles on the surface. The prepared metal surface is composed of silver nanostructures, which present a relatively uniform morphology. Studies have shown that these silver nanostructures have a strong enhancement effect on fluorophores, as shown in Figure 19 and Figure 20. Compared with other methods such as vapor deposition and self-assembly techniques, the electrochemical deposition method is very useful for preparing nanostructures with novel morphologies and specific characteristics, and it is economical, efficient, and easy to use [187,188,189]. Since then, electrochemical preparation of metal substrates has also been widely used. For example, metal nano-fractal structures [190] and metal nano-dendrite-like nano-structures [191] prepared by electrochemical methods have been found. According to experiments, the probe molecules deposited on the surface of the silver fractal nano-structured substrate achieved simultaneous enhancement of fluorescence and Raman signals [191]. It has played a very good experimental support for the research of surface-enhanced spectroscopy. In 2012, Zheng et al. [192] used displacement reaction between a high-purity aluminum substrate and silver nitrate to prepare a flower-like silver nanostructure substrate. Under the action of the substrate, the fluorescence radiation of Rhodamine 6G molecules was enhanced. Dong et al. [193] tried to use the replacement reduction method to prepare Ag/Au bimetallic nano-clusters on polished copper wafers, achieving up to 8 times of the fluorescence enhancement effect. Studies showed that metal nanoparticles in a low temperature environment are more conducive to becoming fluorescent molecules so as to achieve fluorescence radiation enhancement [194]. Generally speaking, the dielectric function of a metal mainly depends on the size of the metal and local environment. The non-free electrons of the metal dielectric function have a more inhibitory effect on the fluorescence quenching at a low temperature environment than at room temperature. However, there is a competitive process between enhancement and quenching. If the quenching process is inhibited, the fluorescent species as a whole exhibit the effect of fluorescence radiation enhancement.

In terms of practical applications, surface-enhanced spectroscopy, as a non-destructive testing technology, has been successfully applied in many fields due to its advantages of high sensitivity, high selectivity, and low cost. Compared with traditional fluorescence spectroscopy detection, metal nanostructures can greatly improve the performance of probes under specific conditions. Related research results have been preliminarily applied to the optical imaging of cancer cells [195] and DNA detection [196], etc.

#### 4.2.4. Optical Applications of Fano Resonance

Surface plasmon resonance in metal nanostructures has rich and unique optical properties and is widely used in fields such as physics, chemistry, materials, and biological sensing, etc. In the 1920s, N. Bohr explored the Rydberg spectrum curve in hydrogen atoms and calculated the atomic structure mode [197]. Later, U. Fano applied quantum mechanics to theoretically explain the discovery of Beutle in the Rydberg spectrum curve. The reason for the existence of the shape of the anti-symmetrical line, the Beutler–Fano formula was proposed to predict this line shape. Subsequently, the Fano theory was widely used in many fields such as atomic and molecular physics. In 1998, T.W. Ebbesen found that the sub-wavelength aperture in the thin metal film had an abnormal light transmission performance. The nonlinear optical effect Fano resonance makes the metal nanostructure have better optical properties such as greater near-field enhancement, higher refractive index sensitivity, and a narrow half-height of spectral resonance peak. Since then, the research on the optical properties of metal nanoparticles has become a research hotspot in the nanophotonics field.

Plasma clusters can support Fano resonance and the cluster’ linear characteristics are controlled by the cluster geometry. In 2012, J. Britt Lassiter and others of the Rice University Nanophotonics Laboratory designed a semicircular-centered nanostructure surrounded by rings of tightly-spaced coupled nanodiscs. When this structure was incorporated with a unique broadband liquid crystal device geometry, the entire Fano resonance spectrum can be switched on and off in a voltage-dependent manner [198]. The application of a relative (~6V) voltage caused a reversible transition between Fano-like and non-Fano-like spectra, resulting in a complete on/off switch of the transparent window. In the same year, Krishnan Thyagarajan and others of the Swiss Federal Institute of Technology proved the significant increase in the second harmonic generation of the Fano resonance in the plasma heptamer made of silver, theoretically and experimentally [199]. This geometric structure is designed to simultaneously generate Fano resonance at the fundamental wavelength, resulting in a strong localization of fundamental field close to the system, and higher-order scattering peaks at the second harmonic wavelength. These results illustrated the versatility of the Fano resonant structure to design specific optical responses in the linear and nonlinear regions, paving the way for future research on the role of dark mode in nonlinear and quantum optics.

Structural coloring is an interference phenomenon. Colors appear when visible light interacts with nanostructured materials. In 2014, Shen et al. [200] of the Massachusetts Institute of Technology proposed a new structural color generation mechanism, which generates color on a thin photonic crystal plate through Fano resonance effect. The proposed concept is realized by experimentally fabricating samples that show colors caused by resonance and have a weak dependence on the viewing angle. The results showed that by stretching the photonic crystal plate fabricated on an elastic substrate, the colors caused by resonance can be dynamically tuned. In 2018, Hwang et al. [201] experimentally observed and theoretically explained the influence of Fano resonance on optical chirality of planar plasmonic nanodevices in the visible wavelength range. The disk-centered nanodevice is surrounded by six gold nanorods, and its orientation angle exhibits optical chirality under dark field illumination. The chiral response induced by gold nanorods is affected by nanodisks of different diameters, which leads to Fano resonances with different coupling strengths. By observing and understanding the influence of Fano resonance on optical chirality, the chiral characteristics of planar subwavelength nanodevices can be effectively manipulated.

**Figure 21:**
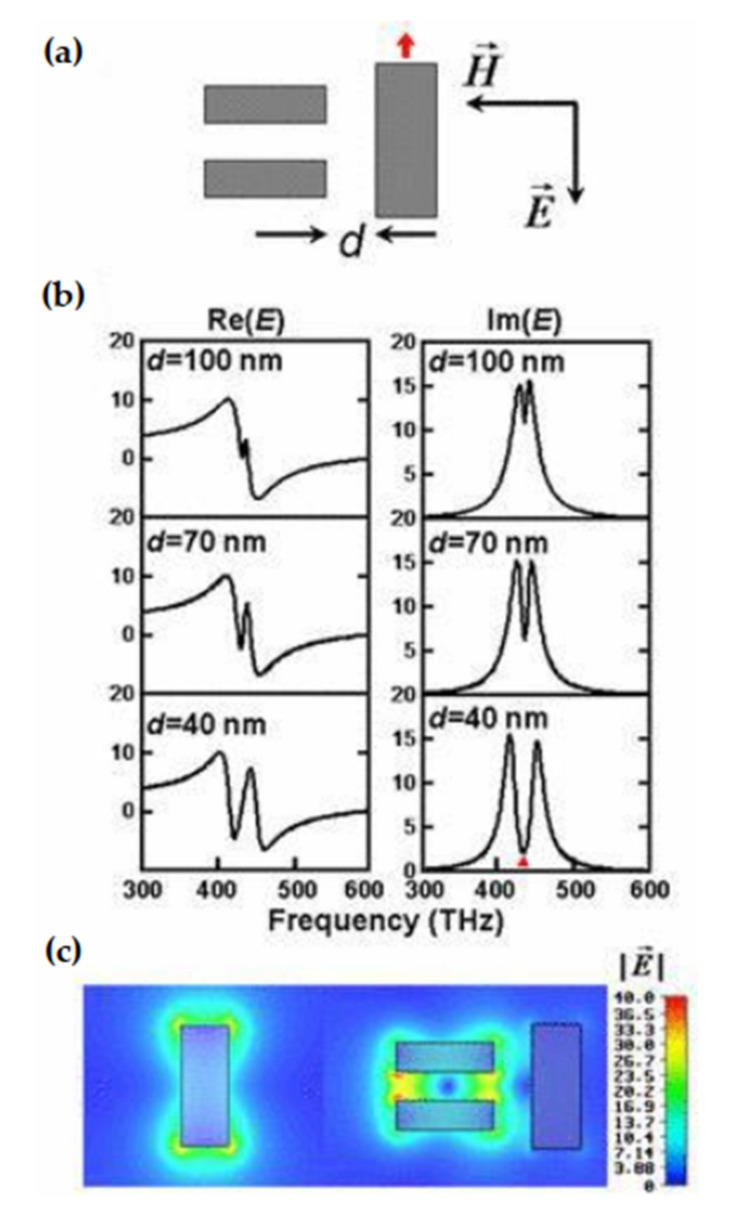
(**a**) Top view of the plasmonic system consisting of a radiative element and a dark element with separation d, with light incident at the normal direction. (**b**) The real part and imaginary part of an E_x_ probe placed at 10 nm from the end facet of the radiative antenna red arrow in (**a**) for separations ranging from 40 to 100 nm between the radiative and dark elements. (**c**) The 2D field plot of an uncoupled radiative atom (**left**) and radiative atom coupled with a dark atom with a separation of 40 nm (**right**) at a frequency of 428.4 THz, as indicated by the red triangle in (**b**). Reprinted with permission from Ref. [202] Copyright 2008 American Physical Society.

Zhang et al. [202] proposed a stone table structure composed of two parallel nano metal rods and a single nano metal rod perpendicular to it. This structure has a single composition, and only uses precious metal gold and surrounding media. In addition, it is simple to make experimentally. More importantly, it supports the Fano resonance very well and obvious asymmetric line patterns can be observed in the spectrum (as shown in Figure 21). The dipole resonance mode of the single rod can be directly activated by the vertically-incident light (the electric field direction is parallel to the rod) and become a bright state. The quadrupole resonance mode of the double rod is a dark state, which needs to be coupled with the dipole resonance mode of the single rod to be activated. When two structures are combined in the same plane to form the stone table structure, the dark mode of double rod needs to be coupled with the bright mode of the single rod in the near field to be activated. Then the dark and bright modes’ interaction formed Fano resonance of the whole structure.

**Figure 22:**
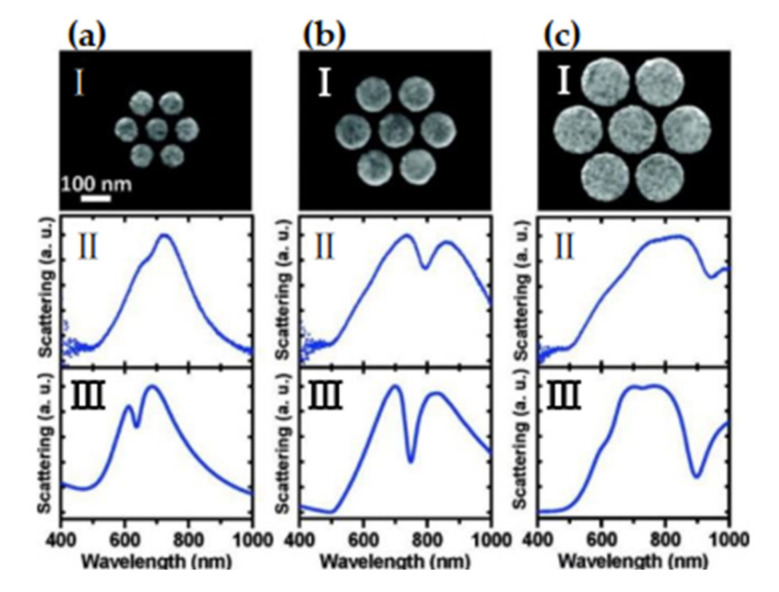
Size dependence of the scattering spectrum of a heptamer: (**a**) 85-nm diameter constituent particles; (**b**) 128-nm diameter particles; and (**c**) 170-nm diameter particles. In all cases, the gap sizes between the particles in the heptamers were nominally ∼15 nm. (**I**) SEM images obtained using an FEI Quanta 400 SEM; (**II**) experimentally obtained dark-field scattering spectra, obtained with unpolarized light, of each individual cluster shown in (**I**); and (**III**) FDTD calculations of the dark-field spectral response of the same structure. Reprinted with permission from Ref. [203]. Copyright 2010 American Chemical Society.

The cluster structure composed of seven nanodiscs designed by Halas et al. [203] can well support the Fano resonance phenomenon, as shown in Figure 22. This structure has the characteristic that the Fano resonance effect is particularly sensitive to changes in the refractive index of the surrounding environment. In addition, it is a cluster structure with very high sensitivity.

The formation mechanism of surface plasmon Fano resonance is analyzed and explained in depth by studying three typical metal nanostructures that can produce Fano resonance, such as symmetry destruction, nanoclusters, and nanoarrays. These typical metal nanostructures have many potential applications in biological detection, surface-enhanced Raman scattering, and nano-optoelectronic devices.

1. Biological detection sensor.

The detection of small changes in the wavelength position of localized surface plasmon resonance in metal nanostructures has been successfully applied in detections such as biological markers. However, practical applications often suffer from the large spectral width of plasmon resonance caused by large radiation damping in the metal nanocavity. Through the custom design and use of repeatable nanofabrication processes, high-quality planar gold plasma nanocavities with strongly-reduced radiation damping are fabricated, and the additional substrate etching leads to a substantial increase in the sensing volume and subsequent increase of sensitivity. Niels Verellen et al. of the University of Luhan, Belgium designed a nanostructure that combines nanocrossbars and nanorods, using the coherent combination of bright and dark plasma modes in nanocrossrods and nanorods to generate high quality factor subradiation Fano resonance [204]. After experiments and other tests, the sensitivity of the structure to the refractive index can be as high as 1000 nm/RIU. Moreover, by further fine-tuning geometric parameters of the nanostructures, the sensing performance and quality factors can be further improved, making these nanocross-systems a valuable platform for biochemical sensing applications and plasma laser emission.

2. Optoelectronic devices.

Electrically-driven plasmon devices can provide unique opportunities as research tools and practical applications. In such a device, the current flowing through the metal tunnel junction excites plasmons, thereby generating light emission. The plasmon generated at the tunnel junction is equivalent to feeding back a plasmon antenna at its source. In this case, it is different from conventional optical excitation of plasma that combines far-field illumination to a plasma antenna and then couples out. This local characteristic of excitation easily enters the evanescent (or “dark”) mode, which is not easily activated by far-field illumination. From a more practical viewpoint, this device can pave the way for the realization of on-chip optical communication and sensing. Yuval Vardi et al. from the Department of Condensed Matter Physics of the Israel Institute of Science proposed an electrically-driven plasmon device that consists of gold nanoparticles trapped in the gap between two electrodes [205]. In several devices, they found a sharp asymmetric tilt angle in the frequency spectrum, and finally determined it as Fano resonance. The calculation of finite difference time domain showed that this resonance was caused by the interference between the nanoparticle and electrode dipole field, and this resonance is easily controlled by structural parameters. The Fano resonance generated in the plasmonic structure of metal nanoparticles has always attracted attention, especially its sharp spectral characteristics and sensitivity to sample parameters, which may make them highly efficient sensors. Therefore, the ability to generate such resonances in electric-driven equipment may amplify their potential. In addition, separate control of wide and narrow mode disturbances in these devices provides a simple way to design Fano resonance. This device has an important reference value for the realization of on-chip controllable nano-optical emitters and sensors.

3. Chemical applications.

Molecular electronics is a new tool for studying quantum transport phenomena. It can be used for third-generation DNA sequencing using nanopores and nanochannels [206]. Some nucleobases in DNA often exist in the form of methylation associated with epigenetic modification to turn on/off the expression of genes or cancer states. Therefore, methylated nucleobases need to be identified and distinguished from normal nucleobases [207]. Nevertheless, molecular electronics is still in its infancy. Therefore, most of the molecular electronics methods for sequencing proposed so far need to be greatly upgraded for practical implementation. For single-molecule spectroscopy and rapid DNA sequencing at the level of atomic resolution, the new concept of two-dimensional molecular electronic energy spectroscopy (2D MES) can be driven by the Fano resonance of bias and gate voltage [208]. This method can analyze molecular fingerprints in the form of Fano resonance rather than in the form of electron transmission, i.e., 2D conduction (high spatial resolution) rather than optical or photonic methods (very low spatial resolution). In order to obtain this Fano resonance of molecules with discrete molecular orbital (MO) energy levels, graphene and graphene nanoribbons (GNR) have become ideal materials with continuous band structures because of the extraordinary electronic characteristics of nanoelectronics. Arunkumar Chitteth Rajan et al. of the National Institute of Science and Technology in Ulsan, South Korea displayed Fan by applying external disturbance to a small GNR (AGNR), 2D MES [209] driven by resonance. Once a molecule is attached to the AGNR surface, the ballistic electron transfer mode can be replaced by the new path of the Fano resonance mode under appropriate resonance conditions. In this way, characteristic 2D differential conductance maps can be used to identify adsorbed molecules and their conformations.

#### 4.2.5. Other Apps

As mentioned earlier, metal plasma nanostructures are materials with potential applications. Many new applications have inspired people to explore the structure of various metal materials [210]. Such as lasers, hyperlenses, active plasmas, optoelectronics [211], phase change materials [212], and polymers [213], etc.

## 5. Conclusions

The continuous research of nanomaterials is not only the embodiment of the rapid development of science and technology, but also the requirements of the development of the times. The development of new materials affects and brings great convenience to human lives. The controllable and low-cost preparation of metal nanostructures have always been a problem in scientific research. In this article, we reviewed the manufacturing methods of metal nanostructures and the applications of metal nanostructures, focusing on the dependence of these applications on the properties of specific nanostructures. The main highlight of this review is that preparation methods of metal nanostructures are classified, and metal nanostructure materials are prepared by these variety of preparation methods into different microstructural characteristics and properties, which can be applied to different fields. Commonly-used characterization methods of metal nanostructures are summarized, which provides an important reference for researchers on the future study of metal nanostructure materials.

The existing metal nanostructure preparation methods are applicable in laboratory research, but there are still some challenges for metal nanostructures to move from laboratory research to widespread applications. For example, the template method can only be applied in mold metal glass, polymers, etc. The chemical synthesis method has the characteristics of high efficiency, but is subjected to the limitation of the type of material and the challenges of controllability. Photolithography technology is capable to prepare most metal structures, however it always produces a high cost and is difficult to prepare nanostructures with a high aspect ratio. The preparation of metal nanostructures still has challenges in terms of high efficiency, controllability (such as resolution, accuracy, and uniformity), material diversity, low cost, and high aspect ratio structure.
